# Supplementary material for: Crawling and Gliding: A Computational Model for Shape-Driven Cell Migration
Source: PLoS Comput Biol. 2015 Oct 21;11(10):e1004280. doi: 10.1371/journal.pcbi.1004280 (PMC4619082; doi:10.1371/journal.pcbi.1004280)
Supplement: S1 Code — (ZIP) [file pcbi.1004280.s012.zip › release/tst/doc/html/parse_8h_source.html]

Tissue Simulation Toolkit: parse.h Source File


Tissue Simulation Toolkit
 0.1.4.1


- Main Page
- Namespaces
- Classes
- Files

- File List
- File Members

parse.h

Go to the documentation of this file.

1 /\*

2

3 Copyright 1996-2006 Roeland Merks

4

5 This file is part of Tissue Simulation Toolkit.

6

7 Tissue Simulation Toolkit is free software; you can redistribute

8 it and/or modify it under the terms of the GNU General Public

9 License as published by the Free Software Foundation; either

10 version 2 of the License, or (at your option) any later version.

11

12 Tissue Simulation Toolkit is distributed in the hope that it will

13 be useful, but WITHOUT ANY WARRANTY; without even the implied

14 warranty of MERCHANTABILITY or FITNESS FOR A PARTICULAR PURPOSE.

15 See the GNU General Public License for more details.

16

17 You should have received a copy of the GNU General Public License

18 along with Tissue Simulation Toolkit; if not, write to the Free

19 Software Foundation, Inc., 51 Franklin St, Fifth Floor, Boston, MA

20 02110-1301 USA

21

22 \*/

23

24 char \*ParsePar(FILE \*fp, char \*parameter, bool wrapflag);

25 int igetpar(FILE \*fp,char \*parameter, bool wrapflag);

26 int igetpar(FILE \*fp,char \*parameter, int default\_val, bool wrapflag);

27 float fgetpar(FILE \*fp,char \*parameter, bool wrapflag);

28 float fgetpar(FILE \*fp,char \*parameter, double default\_val, bool wrapflag);

29

30 /\* Get a list of n comma separated doubles \*/

31 double \*dgetparlist(FILE \*fp,char \*parameter, int n, bool wrapflag);

32 char \*sgetpar(FILE \*fp,char \*parameter, bool wrapflag);

33 char \*sgetpar(FILE \*fp,char \*parameter,const char \*default\_val, bool wrapflag);

34 bool bgetpar(FILE \*fp, char \*parameter, bool wrapflag);

35 bool bgetpar(FILE \*fp, char \*parameter, int default\_val, bool wrapflag);

36 char \*SearchToken(FILE \*fp, char \*token, bool wrapflag);

37 int TokenInLineP(char \*line,char \*token);

38 void SkipToken(FILE \*fp,char \*token, bool wrapflag);

39 void SkipLine(FILE \*fp);

40 char \*bool\_str(bool bool\_var);

41

bool\_str

char \* bool\_str(bool bool\_var)

**Definition:** parse.cpp:222

TokenInLineP

int TokenInLineP(char \*line, char \*token)

**Definition:** parse.cpp:362

igetpar

int igetpar(FILE \*fp, char \*parameter, bool wrapflag)

**Definition:** parse.cpp:69

fgetpar

float fgetpar(FILE \*fp, char \*parameter, bool wrapflag)

**Definition:** parse.cpp:100

bgetpar

bool bgetpar(FILE \*fp, char \*parameter, bool wrapflag)

**Definition:** parse.cpp:238

dgetparlist

double \* dgetparlist(FILE \*fp, char \*parameter, int n, bool wrapflag)

**Definition:** parse.cpp:136

SearchToken

char \* SearchToken(FILE \*fp, char \*token, bool wrapflag)

**Definition:** parse.cpp:288

ParsePar

char \* ParsePar(FILE \*fp, char \*parameter, bool wrapflag)

**Definition:** parse.cpp:34

SkipLine

void SkipLine(FILE \*fp)

**Definition:** parse.cpp:371

sgetpar

char \* sgetpar(FILE \*fp, char \*parameter, bool wrapflag)

**Definition:** parse.cpp:189

SkipToken

void SkipToken(FILE \*fp, char \*token, bool wrapflag)

**Definition:** parse.cpp:380


---

Generated on Thu Aug 14 2014 22:04:01 for Tissue Simulation Toolkit by  

 1.8.6
